# PCM4EU and PRIME-ROSE: Collaboration for implementation of precision cancer medicine in Europe

**DOI:** 10.2340/1651-226X.2024.34791

**Published:** 2024-05-23

**Authors:** Kjetil Taskén, Soemeya F. Haj Mohammad, Gro Live Fagereng, Ragnhild Sørum Falk, Åslaug Helland, Sahar Barjesteh van Waalwijk van Doorn-Khosrovani, Katarina Steen Carlsson, Bettina Ryll, Katriina Jalkanen, Anders Edsjö, Hege G. Russnes, Ulrik Lassen, Ebba Hallersjö Hult, Iwona Lugowska, Jean-Yves Blay, Loic Verlingue, Edvard Abel, Maeve A. Lowery, Matthew G. Krebs, Kristoffer Staal Rohrberg, Kristiina Ojamaa, Julio Oliveira, Henk M.W. Verheul, Emile E. Voest, Hans Gelderblom

**Affiliations:** a Institute for Cancer Research, Oslo University Hospital and Institute of Clinical Medicine, University of Oslo, Oslo, Norway; b Department of Medical Oncology, Leiden University Medical Centre, Leiden, The Netherlands; c Oslo Centre for Biostatistics and Epidemiology, Oslo University Hospital, Oslo, Norway; d Division of Cancer Medicine, Oslo University Hospital, Oslo, Norway; e CZ Health Insurance, Tilburg, The Netherlands; f Swedish Institute for Health Economics, Lund, Sweden; g Stockholm School of Economics Institute for Research, Stockholm, Sweden; h Clinical Trial Unit, Comprehensive Cancer Centre, Helsinki University Hospital, Helsinki, Finland; i Department of Clinical Genetics, Pathology and Molecular Diagnostics, Region Skåne, Malmö, Sweden; j Department of Pathology, Oslo University Hospital, Oslo, Norway; k Department of Oncology, Rigshospitalet, Copenhagen University Hospital, Copenhagen, Denmark; l Maria Sklodowska-Curie Institute of Oncology, Warsaw, Poland; m Centre Léon Bérard, Lyon, France; n Department of Oncology, Institute of Clinical Sciences, Sahlgrenska Academy at University of Gothenburg, Gothenburg, Sweden; o Department of Oncology, Sahlgrenska University Hospital, Gothenburg, Sweden; p Trinity St James Cancer Institute, Trinity College, Dublin, Ireland; q Division of Cancer Sciences, The University of Manchester and The Christie NHS Foundation Trust, Manchester, UK; r Hematology-Oncology Clinic at Tartu University Hospital, Tartu, Estonia; s Instituto Português de Oncologia do Porto FG, Porto, Portugal; t Department of Medical Oncology, Erasmus MC Cancer Institute, Rotterdam, The Netherlands; u Department of Molecular Oncology & Immunology, Netherlands Cancer Institute, Amsterdam, The Netherlands

**Keywords:** DRUP-like clinical trials, targeted drugs, reimbursement, public-private collaboration, synthetic control arms, molecular tumour boards, implementation

## Abstract

**Background:**

In the two European Union (EU)-funded projects, PCM4EU (Personalized Cancer Medicine for all EU citizens) and PRIME-ROSE (Precision Cancer Medicine Repurposing System Using Pragmatic Clinical Trials), we aim to facilitate implementation of precision cancer medicine (PCM) in Europe by leveraging the experience from ongoing national initiatives that have already been particularly successful.

**Patients and methods:**

PCM4EU and PRIME-ROSE gather 17 and 24 partners, respectively, from 19 European countries. The projects are based on a network of Drug Rediscovery Protocol (DRUP)-like clinical trials that are currently ongoing or soon to start in 11 different countries, and with more trials expected to be established soon. The main aims of both the projects are to improve implementation pathways from molecular diagnostics to treatment, and reimbursement of diagnostics and tumour-tailored therapies to provide examples of best practices for PCM in Europe.

**Results:**

PCM4EU and PRIME-ROSE were launched in January and July 2023, respectively. Educational materials, including a podcast series, are already available from the PCM4EU website (http://www.pcm4eu.eu). The first reports, including an overview of requirements for the reimbursement systems in participating countries and a guide on patient involvement, are expected to be published in 2024.

**Conclusion:**

European collaboration can facilitate the implementation of PCM and thereby provide affordable and equitable access to precision diagnostics and matched therapies for more patients.

## Introduction

**Figure 1:**
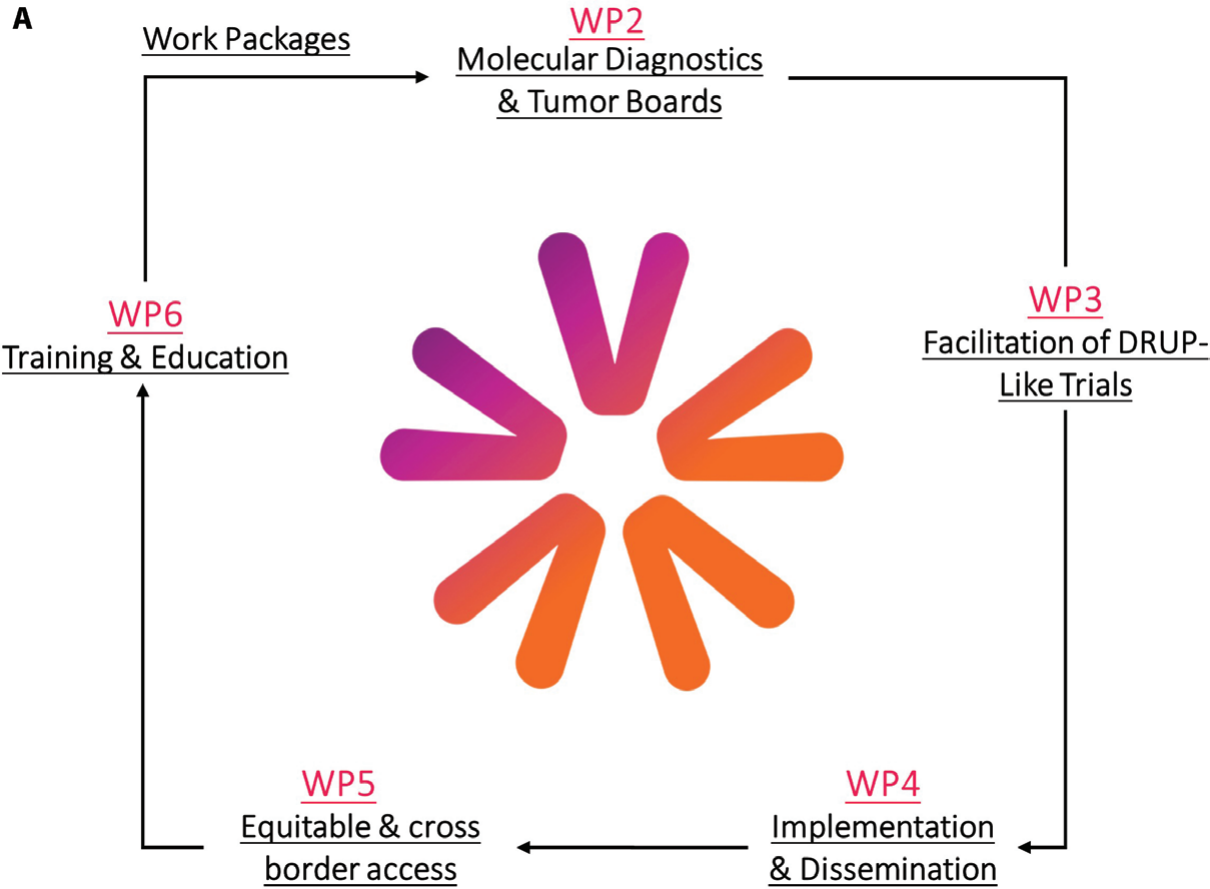
Overview of the planned work-packages. (A) Work packages in PCM4EU. H. Gelderblom (PCM4EU coordinator) is leading WP1, A. Edsjö and H. Russnes are leading WP2, U. Lassen and Å. Helland are leading WP3, E. Hult and K. Taskén are leading WP4, B. Ryll and U. Lassen are leading WP5, and I. Lugowska and J.Y. Blay are leading WP6. (B) Work packages in PRIME-ROSE. H. Gelderblom is leading WP1, R. Falk is leading WP2, Å. Helland is leading WP3, S.B van Waalwijk van Doorn-Khosrovani is leading WP4, K. Carlsson is leading WP5, B. Ryll is leading WP6, K. Taskén (PRIME-ROSE coordinator) is leading WP7, K. Jalkanen is leading WP8 whereas Charlotte J. Haug is the independent ethics review in WP9.

In recent years, evidence on the role of multigene sequencing in improving outcomes in patients with metastatic cancer has been widened, reinforcing the importance of precision medicine [1–3]. Several precision cancer medicine (PCM) initiatives have demonstrated the feasibility and benefits of implementing PCM within individual countries. PCM4EU (Personalized Cancer Medicine for all EU citizens) (http://www.pcm4eu.eu/) and PRIME-ROSE (Precision Cancer Medicine Repurposing System Using Pragmatic Clinical Trials) (http://www.prime-rose.eu/), are two projects funded through the EU4Health and Horizon Europe’s EU Mission on Cancer programmes. The projects are built on the successes of national initiatives centred around Drug Rediscovery Protocol (DRUP)-like clinical trials (DLCTs), intending to expand equitable and sustainable access to PCM for more patients by addressing key challenges related to implementation (see Figure 1).

**Figure 2:**
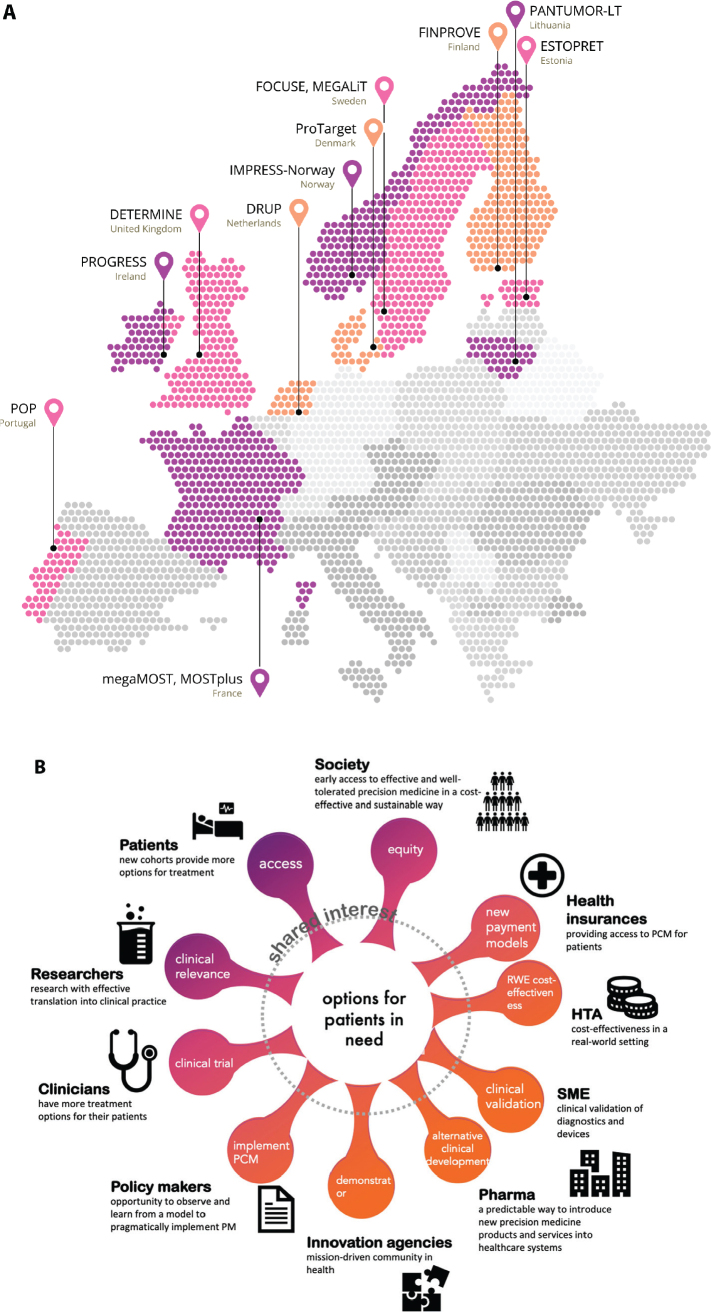
Overview of all ongoing and soon-to-start DRUP-like trials in Europe. (A) DRUP in the Netherlands, PIs E.E Voest, H. Gelderblom & H.M.W. Verheul, ProTarget in Denmark, PIs K.S. Rohrberg & U. Lassen. FINPROVE in Finland, PI K. Jalkanen, IMPRESS-Norway in Norway, PI Å. Helland, MOSTplus and megaMOST in France, PIs J.Y. Blay & L. Verlingue, DETERMINE in United Kingdom, PI M.G. Krebs, POP in Portugal, PI J. Oliveira, ESTOPRET in Estonia, PI K. Ojamaa, and PANTUMOR-LT in Lithuania, PI E. Baltruškevičienė. (B) Stakeholder involvement in PRIME-ROSE.

The success of the original DRUP-trial from the Netherlands, showing high inclusion rate and clinical benefit (CB), inspired similar trials in other countries [4]. All involved national trials are based on or aligned with DRUP (PIs Emile Voest, Hans Gelderblom, Henk Verheul) [5, 6], while being independently organised, governed and financed (Table 1). Such trials are ongoing or soon to be initiated in 11 European countries (Figure 2A), with more countries preparing to launch DLCTs. DRUP is also collaborating with the American TAPUR Study and CAPTUR in Canada, that are built on the same principles as the European trials [7, 8], and has aggregated data with the Australian MoST trial [9].

**Table 1:** Overview of overlapping primary and secondary endpoints in the DRUP-like clinical trials.

Endpoint in DRUP-like clinical trials	DRUP	ProTarget	IMPRESS	FINPROVE	MOSTplus	DETERMINE	POP	FOCUSE	ESTOPRET
**Disease control at 16 weeks after treatment initiation**	X	X	X	X	X	X^*^	X	X	X
**Progression-free and overall survival**	X	X	X	X	X	X	X	X	X
**Duration of treatment on study (time on drug)**	X	X	X	X	X	X	X	X	X
**Treatment related grade ≥3 and SAE**	X	X	X	X		X	X	X	X
**Objective tumour response**	**X**	**X**	**X**		**X**	**X**	**X**	**X**	
**% of patients treated based on their molecular profile**	**X**	**X**	**X**		**X**		**X**	**X**	**X**

An overview of overlapping main endpoints in the ongoing DRUP-like clinical trials.

* Durable Clinical Benefit, defined as the absence of disease progression for at least 24 weeks from the start of trial treatment. Dark blue = 100% similarity across trials, blue = 90% similarity across trials, and light blue = 80% similarity across trials.

DLCTs are prospective phase II combined umbrella-basket trials in which patients with advanced cancers receive targeted therapies matched to genomic alterations in their tumour. Each trial enrols participants into defined cohorts, based on the combination of tumour type, molecular alteration, and targeted therapy, with CB as the primary endpoint. Common for the DLCTs is that they combine accessibility with affordability by providing broad access to PCM for patients who have exhausted all other standard treatment options. The trials continuously generate evidence that can be linked to pragmatic outcome-based reimbursement schemes, thereby enabling reimbursement when the necessary evidence (e.g. the PASKWIL criteria in the Netherlands) has been gathered [10, 11]. An overview of on-label and off-label reimbursement systems in Europe is also part of the deliverables in PRIME-ROSE.

The projects will provide recommendations regarding the use of next-generation sequencing (NGS)-panels and clinical decision support system (CDSS)-tools, together with detailed guidelines for molecular diagnostics in the cancer care pathway to facilitate implementation of PCM in additional countries. Furthermore, novel methods for establishing relative effectiveness, as well as strategies for evaluation of cost-effectiveness of PCM, will be developed. This will be used to facilitate access to new treatment options.

## Advancing Molecular Diagnostics in PCM

PCM4EU aims to facilitate implementation of adequate molecular diagnostics into standard-of-care for all patients in Europe (Figure 1A). Clinical molecular diagnostic assays must detect all relevant genetic variants for adequate therapy decisions, while simultaneously identifying patients for clinical trials. The rapid development of new classes of targeted therapies and the promising results of immunotherapy, both necessitating use of more complex biomarkers, has resulted in a growing need for tools to guide in choosing NGS-based assays and CDSS tools.

To address this challenge, PCM4EU is gathering data on NGS-based assays used by the participating centres to create a curated database containing information on gene panel content in relation to targeted therapy and clinical trial inclusion criteria. Several of the DLCTs use both NGS and whole genome sequencing (WGS) as part of the diagnostic work-up. Based on available data, *in-silico* comparisons will be performed on commonly used NGS-panels versus WGS, and the added value of introducing WGS will be evaluated through health technology assessment. Real-life and synthetic large-scale datasets will be developed to harmonise the interpretation of key complex biomarkers such as microsatellite instability (MSI), tumour mutational burden (TMB), and homologous recombination deficiency (HRD).

PCM4EU will map out currently available CDSS tools for clinical decision-making in oncology. Evaluation will include feature mapping, tool requirements, integration options, compatibility with requirements for patient security, and *In Vitro* Diagnostic Regulation status. To expand and structure knowledge on available CDSS tools, including artificial intelligence-based tools, these tools and their knowledge management strategies will be charted. Moreover, the project will develop a conceptual framework for performance testing across multiple cancer-relevant features beyond the core variant characterisation, like single nucleotide variants and insertions and deletions, including copy-number variations, selected complex biomarkers (i.e., TMB, HRD, MSI), neoantigens, transcription profiles and specific gene signatures (e.g. immune signatures), and gene fusions. Finally, we plan to evaluate the performance of clinical trial matching across CDSS platforms to improve harmonisation between different recommendations by enhancing the accuracy of available clinical trial data and mapping the identification and definition of actionable targets [12].

## Initiation of DLCTs in Europe

The PCM4EU and PRIME-ROSE projects will support countries without DLCTs in setting up trials in countries where they are not yet available by sharing current protocols, standard operating procedures, electronic case report forms (eCRFs), and ways for harmonised data collection (Figure 1). Each DRUP-founder country will co-create efficient knowledge transfer to centres in the start-up phase.

For joint data analyses across DLCTs, the key primary endpoint for the combined analyses is uniformly implemented across the trials and defined as CB, meaning a confirmed stable disease, partial or complete response at 16 weeks after treatment initiation (Table 1). A formal data sharing protocol has been developed and agreed upon.

## Access to New Therapies: Single Point Access to Multi-Trial Network

Data sharing and aggregation between the trials will allow for monitoring of cohort recruitment from each trial and cross-trial evaluation of cohorts when recruitment is complete, increasing the inclusion rate. Moreover, data sharing will facilitate collaboration with the pharmaceutical industry. Pharmaceutical companies can choose to access all trials simultaneously with their drugs through PRIME-ROSE, providing a single point of entry, where pharma partners provide free drugs and floating treatment slots to the consortium. This approach will significantly reduce time for initiation and rapidly increase inclusion rates while removing a major barrier for establishing a DLCT, since access to drugs is critical for trial initiation.

## Establishment of Relative Effect: Innovative Models for Synthetic Control Cohorts

DLCTs are actively searching for patients with defined target mutations to treat them with the specified drugs. Randomisation for treatment allocation after progression on standard-of-care is not advised due to the rarity of driver genomic alterations and the lack of treatment alternatives. To establish relative efficacy, multiple synthetic control cohorts will be built to reduce bias and resembling a randomised controlled trial.

PRIME-ROSE will establish three types of control cohorts, based on available data from registries and sequenced cohorts that have received standard-of-care therapy: 1) Patients with a known genomic alteration that received standard treatment. However, as specific molecular alterations will have a low prevalence, these control cohorts might be small. 2) Patients without a targetable genomic alteration. The characteristics of patients with and without genomic alterations will be compared to find patterns associated with specific targets and calculate the expected probability of the alterations. 3) Extraction of larger patient control cohorts from registry data. For these cohorts, information about the presence or absence of a specific mutation will not necessarily be available, but the frequency of the mutation in the population may be known. Therefore, based on the probability of the target mutation, randomisation will be performed to synthetic control arms to reduce selection bias.

## Implementation into Standard-of-Care

Whereas regulatory approval is centralised to the European Medicines Agency, reimbursement systems are country-specific, with varying requirements in terms of data. To facilitate implementation, PRIME-ROSE will identify requirements of reimbursement systems in participating countries, including systems for off-label reimbursement. A model for economic evaluation will be designed and constructed, including budget impact and cost-effectiveness analysis of PCM, integrating the chain of decision from molecular diagnostics to treatment. Information collected on local requirements for reimbursement assessment will be used to ensure that conducted analyses will meet the criteria for decision-making in different countries. Furthermore, collected data will provide a background to evaluate and compare how differences in reimbursement systems can impact access, timing and affordability.

## National Guidelines for Precision Diagnostics

To facilitate the development of national guidelines for precision diagnostics, the PCM4EU consortium will develop a best practice on which cancer patients should be offered molecular diagnostics and what should be included in the molecular diagnostic work-up as part of standard-of-care. This includes recommendations on selecting the most appropriate molecular assays and how to process data to match results with anti-cancer therapies according to up-to-date evidence (in accordance with European Society for Medical Oncology (ESMO) clinical guidelines [13]). The guidelines will focus on the added value of using advanced diagnostic tests in terms of higher precision in choosing treatment strategies as well as costs and value of implementation. We aim to include costs from a wide societal perspective to demonstrate broader impact. However, results will be reported disaggregated to allow for interpretations from more narrow perspectives preferred in some countries.

## Risk-Sharing Agreement and Access to Therapies

Participants in pivotal trials are usually not representative of patients encountered in standard clinical practice, especially since rare tumour types are often not represented. Here, we face the ragged edges of clinical practice, where off-label use is common since there are no alternative treatment options, the effectiveness and safety data are often not collected, the practice is not harmonise or regulated, and there are disparities and inequalities in access. A comprehensive approach to this clinical reality can stimulate repurposing drugs to offer safe, effective, and affordable treatment options for the diverse cancer patient population commonly seen in clinical practice.

To evaluate the effectiveness of a treatment in routine clinical practice, real-world data will be systematically collected. Such practice is carried out by the DRUG Access Protocol (DAP) in the Netherlands [14]. DAP is a pragmatic, non-randomised protocol that prospectively collects effectiveness and toxicity data on novel authorised or unauthorised anticancer therapies awaiting regulatory approval and reimbursement, but also authorised anticancer therapies that are not being reimbursed for an on-label or off-label indication due to data gaps.

In PRIME-ROSE, strategies for pragmatic outcome-based risk-sharing agreements will be explored by identifying factors that were critical for the successful establishment of such agreements in the Netherlands and Norway [15]. Based on a mapping of the varying requirements as regards to the implementation of new treatments and expansion to new treatment groups, PRIME-ROSE will assist stakeholders, including national healthcare providers, policymakers, and authorities in EU regions, Member States, and Associated Countries who desire to implement pragmatic reimbursement models for PCM in a real-world setting. The potential for a European framework to evaluate data effectively and efficiently to implement PCM will also be investigated, especially for rare cancers and those with high unmet medical needs. In addition, we will explore the potential of using DRUP-generated evidence to inform regulatory decision-makers at the European level.

## PCM addressing Patient Needs

The patient relevance of the clinical trial endpoints in use will be confirmed to ensure that DLCTs adequately address patients’ needs. Currently, a multi-stakeholder consultation that will inform the selection of health-related quality-of-life endpoints for use across the joint cohorts is in preparation. Furthermore, the stability of patient preferences during and after trial participation will be investigated. Moreover, we are working with a growing community of European cancer patient advocates with expertise in PCM and iterate on existing patient involvement to ensure consistent and meaningful patient involvement throughout the work of the PCM community.

## Successful Multistakeholder Collaborations for Change in Complex Systems

Changes in the healthcare system require participation and interaction between a multitude of stakeholders (Figure 2B). In PRIME-ROSE, we plan to map and describe the PCM ecosystem and develop a PCM theory of change for healthcare as a complex adaptive system with implications for governance, policy, and innovation. We will reflect on differences between the Member States. Building on the concept of Living Labs [16] and the already established practice of peer-to-peer support, the project will develop a ‘DRUP methodology’ to facilitate implementation.

## Conclusion

The PCM4EU and PRIME-ROSE projects strive to reduce inequality in cancer treatment by promoting access to PCM for all European patients through collaborative efforts, patient engagement, and pragmatic outcomes-based approaches.

## Data Availability

No new data were presented in this study. Data sharing is not applicable to this short report.
